# Transcriptome analysis of hepatopancreas of *Eriocheir sinensis* with hepatopancreatic necrosis disease (HPND)

**DOI:** 10.1371/journal.pone.0228623

**Published:** 2020-02-21

**Authors:** Zongying Yang, Kun Hu, Yujie Hou, Yulan Wang, Yi Yao, Xiaoqing Lei, Baohua Yan, Qinglong Jiang, Chunxian Xiong, Liangqing Xu, Liugen Zeng

**Affiliations:** 1 Nanchang Academy of Agricultural Sciences, Nanchang, China; 2 National Pathogen Collection Center for Aquatic Animals, Shanghai Ocean University, Pudong, Shanghai, China; Marquette University, UNITED STATES

## Abstract

Hepatopancreatic necrosis disease (HPND) is a newly emerging disease in the Chinese mitten crab, *Eriocheir sinensis*, which has resulted in large economic losses. However, the underlying cause of this disease remains unclear. To better understand the pathogenesis and pathogenic mechanism of HPND, we compared the transcriptome differences of the hepatopancreas of *E*. *sinensis* with and without HPND. The analysis yielded > 30 million reads for each sample of three test (with HPND) and three control groups (without HPND). We observed 978 downregulated genes and 644 upregulated genes. Among the gene ontology categories “biological process,” “cellular component,” and “molecular function”, the subcategories cellular process, single-organism process, biological regulation, metabolic process, cell part, organelle, organelle part, binding, and catalytic were enriched. Kyoto Encyclopedia of Genes and Genomes (KEGG) pathway analysis showed that “metabolism of xenobiotics by cytochrome P450,” “drug metabolism—cytochrome P450,” “chemical carcinogenesis,” and “material metabolism” were the “five” most significantly enriched pathways in the hepatopancreas of *E*. *sinensis* with HPND. The results revealed that material metabolic abnormalities and drug effects from the external environment might be associated with HPND in the Chinese mitten crab. Considering the wide use of pyrethroids for pond cleaning in Xinghua city, we speculated that pyrethroids might cause HPND in the Chinese mitten crab. Our study provided useful information about the cause and pathogenetic mechanisms of HPND and could help to prevent this disease in production practice.

## Introduction

The Chinese mitten crab, *Eriocheir sinensis*, is one of the most important economic crustacean species in China, and techniques to rear its larvae and its culture facilities were developed in the early 1980s. The Chinese mitten crab is regarded as a delicious and nutritious crustacean by many Chinese consumers because of its nutritional ingredients and delicate flavor [1]. With the increasing demand for the Chinese mitten crab in food markets, the commercial production of *E*. *sinensis* is rapidly expanding. As a province hosting large scale *E*. *sinensis* farming, the annual output of *E*. *sinensis* in Jiangsu Province, China is worth approximately US$ 4 billion [2]. With the development of crab breeding industry, many diseases, such as black gill syndrome, shell disease, edema disease [3], and tremor disease (TD) [4] have emerged and have resulted in serious economic losses. Fortunately, researchers have revealed the etiologies of these diseases and the corresponding prevention and control measures have been developed. However, the disease termed hepatopancreatic necrosis disease (HPND) in *E*. *sinensis* is prevalent in Xinghua city, Jiangsu province, and has spread slowly to other main areas of crab rearing nationwide since 2015 [5]. The clinical symptoms are a color change of the hepatopancreas from orange-yellow to light yellow or gray white, muscle atrophy, and edema. Although the diseased crabs do not die immediately, they are of no economic value.

Despite intensive research into HPND, its etiology has not been elucidated clearly. Ding et al. first claimed that the microsporidian fungus *Hepatospora eriocheir* was the pathogen of HPND in the Chinese mitten crab [5]. They proposed a pathogenic mechanism by which *H*. *eriocheir* starved its host by destroying the hepatopancreas by appropriating host resources, resulting in associated changes in crab metabolism and immunity [6]. However, Pan et al. observed hepatopancreatic cells, spermatogonium, gill tissues, and muscle cells of diseased crabs using transmission electron microscopy, and microorganisms such as bacteria, fungi, microsporidia, and viruses were not detected. In addition, when the healthy crabs were fed or injected with diseased tissues, the symptoms of HPND did not appear. Furthermore, HPND symptoms appeared when crabs were cultured in water with a pH of 9.5 to 10, but not when they were cultured in water with different concentrations of avermectin. Therefore, they concluded that HPND in the Chinese mitten crab was not caused by a virus or microsporidian, but caused by water at a high pH or other environmental factors [7]. Shen et al. [8] constructed the meta-transcriptomic libraries of the hepatopancreas from crabs with and without HPND, and no significant differences in viral and microsporidial communities were detected. However, the prevalence of bacteria belonging to the Tenericutes and Actinobacteria phyla increased, whereas the prevalence of bacteria belonging to the Bacteroidetes phylum decreased in crabs with HPND. In addition, the expression of carboxylesterase family genes was significantly upregulated; therefore, they claimed that HPND was not likely to be caused by a microsporidian and speculated that pyrethroid insecticides injured the hepatopancreas, thus leading to abnormalities in metabolism, nutrient absorption, and microbial dysbiosis; however, this required further study. Gao et al. [9] used liquid chromatography-mass spectrometry to analyze the metabolite profile of the hepatopancreas of crabs with HPND, and found that *Hepatospora eriocheir* was unlikely to be the cause of HPND in the Chinese mitten crab. They revealed that fatty acid metabolic abnormalities and high concentrations of propamocarb might be associated with HPND. Yang et al. [10] sought the etiology of HPND by parasites examination, pathogenic bacteria isolation, challenge experiment, and electron microscopy observation; however, no pathogenic microorganisms were found. The authors suggested that the disease is caused by non-living agents. Generally, there is no unanimous conclusion about the etiology of HPND in the Chinese mitten crab, and all the above speculations require further confirmation.

Gene expression variations in response to external stimuli are sensitive and rapid, and such transcriptional responses might help to explain how an organism responds to a particular situation [11]. Recently, as a highly effective technology to investigate gene expression and to analyze differentially expressed genes and novel transcripts [12], high-throughput RNA-sequencing has been applied widely to study a large variety of invertebrates, such as *Crassostrea gigas* [13], *Litopenaeus vannamei* [14], and *E*. *sinensis* [15, 16].

In crustaceans, the hepatopancreas is a digestive gland and an immune organ, which plays an important role in the metabolism of xenobiotics and the innate immune system [17–19]. Transcriptome analyses of *Litopenaeus setiferus* and *L*. *vannamei* indicated that the hepatopancreas has a vital role in non-specific immunity, and a cDNA library prepared from the hepatopancreas is more representative than that of hemocytes [17]. For the Chinese mitten crabs with HPND, the changes in the color and morphological characteristics of the hepatopancreas are the most important clinical symptoms. Therefore, the present study aimed to analyze the transcriptome of the hepatopancreas of *E*. *sinensis* with and without HPND using Illumina sequencing and bioinformatic analysis. The objective of the study was to annotate functional genes identified using transcriptome analysis, and to evaluate the transcriptomic response of the Chinese mitten crab affected with HPND. Our study will help to understand the etiology and pathogenic mechanism of HPND in *E*. *sinensis* and will provide a reference for further research.

## Materials and methods

### Statement of ethics

This study was carried out in strict accordance with the recommendations and guidelines on the care and use of animals for scientific purposes established by the Institutional Animal Care and Use Committee of Shanghai Ocean University, Shanghai, China. The study design and protocol were approved by the Ethics Committee of Shanghai Ocean University (Approval Number: SHOU-DW-2016-021; SHOU-DW-2019-053). The crabs were anesthetized and dissected on ice; all efforts were made to minimize their suffering.

### Maintenance and treatment of *E*. *sinensis*

Healthy mature male crabs (average weight, 115.26 ± 6.84 g), with yellow hepatopancreas, and diseased mature male crabs (average weight, 113.28 ± 7.26 g), with clinical symptoms of HPND, were collected from the crab breeding base (32.864256110°N, 119.866714447°E) of Tao Huadao Agricultural

Development Co., Ltd. in Anfeng Town, Xinghua City, northern Jiangsu Province, China, in December 2016. These crabs were transported to the laboratory in polystyrene boxes filled with cultivation water, which was aerated during transportation using an aeration pump. Upon arrival, the crabs were anesthetized and dissected on ice to collect the hepatopancreas, which was stored in liquid nitrogen at −80°C for RNA extraction. Healthy crabs and diseased crabs were separated into three groups (six groups in total). Each group consisted of a mixed collection of ten crabs, and the test and control group consisted of three replicates, respectively. A total of 60 crabs were used.

### RNA isolation, cDNA library construction, and sequencing

According to the manufacturer’s instructions, total RNA was isolated from hepatopancreas using the TRIzol reagent (Invitrogen, Waltham, MA, USA). DNA contaminants were removed using RNase-free DNase I (Takara Biotechnology, Dalian, China), and the total RNA was eluted in 100 μL of RNase-free MilliQ H_2_O. The eluted RNA was stored in liquid nitrogen before the next step. A NanoDrop ND-1000 UV–vis Spectrophotometer (NanoDrop Technologies, Wilmington, DE, USA) was used to check the RNA quality. RNA integrity was determined using an Agilent 2100 Bioanalyzer (Agilent Technologies, Palo Alto, CA, USA) and an RNA 6000 Nano LabChip kit (Agilent Technologies). Poly (A) mRNA was isolated using Oligotex mRNA kits (Qiagen) and oligo-dT beads. The mRNA was treated using fragmentation buffer, and the first-strand cDNA was synthesized using the cleaved RNA fragments as templates. RNase H and DNA polymerase I were used to synthesize the second-strand cDNA. A single “A” base was added to these double-stranded cDNA fragments using Klenow 3′–5′ exopolymerase, and the fragments were end-repaired using T4 polynucleotide kinase, T4 DNA polymerase, and the Klenow fragment. Then, the double-stranded fragments were ligated to an index adapter or adapter using T4 quick DNA ligase. Then, the adaptor-ligated fragments were screened out according to their size via agarose gel electrophoresis. The target cDNA fragments were excised from the gel and a PCR reaction was performed to amplify them. After validation using the ABI StepOnePlus Real-Time PCR System (ABI, Foster City, CA, USA) and the Agilent 2100 Bioanalyzer, the cDNA libraries of the corresponding samples were sequenced on a flow cell with the high-throughput mode on an Illumina HiSeq 2500 unit (Illumina, San Diego, CA, USA) [20].

### Data processing and quality control

Trim Galore was used to filter out the low-quality reads and remove 3′ adapter sequences. The FastQC software (http://www.bioinformatics.babraham.ac.uk/projects/fastqc/) was used to clean the obtained reads and to evaluate the quality and content of the remaining clean reads. Then, we conducted a comparative analysis with the reference genome of the Chinese mitten crab. For every sample belonging to the control and test groups, sequence alignment with the reference genome sequences was performed using TopHat (version 2.0.12), with default parameters [21].

### Identification of the differentially expressed genes (DEGs)

The RSEM software with default parameter settings [22] was used to estimate the expression levels of specific transcripts using fragments per kilobase of transcript per million fragments mapped (FPKM) [23]. The expression levels of transcripts in the control and test groups were transformed using base log_2_ (FPKM+1) and evaluated. The DESeq software (version 1.14.0) was used to screen out the DEGs and the fold-change of each transcript was calculated [24]. A two-fold change in expression identified a DEG, and an adjusted *p*-value < 0.05 was considered statistically significant [20].

### GO functional annotation and enrichment analysis for DEGs

To investigate the potential functions of these DEGs, the DEGs were firstly annotated using the UniProt database (http://www.uniprot.org/). The Blast2GO suite (https://www.blast2go.com/) was used to obtain the GO annotations against gene ontology terms (GO; http://www.geneontology.org) [25]. All DEGs were mapped to all GO terms in the GO database, and the number of DEGs mapped to each term was calculated. When compared with the transcriptome background, we used the hypergeometric test to identify the enriched GO terms of the DEGs. The formula used was as follows:

$$P=1-\sum_{i=0}^{m-1}\frac{\left(\begin{array}{l} M\\ i \end{array}\right)\left(\begin{array}{l} N-M\\ n-i \end{array}\right)}{\left(\begin{array}{l} N\\ n \end{array}\right)}$$

where, *N* is the number of genes with GO annotation added; *n* represents the number of DEGs included in *N*; *M* represents the number of genes that were annotated to the specific GO term; and *m* represents the number of DEGs included in *M*. The *p*-value was assigned to each gene and adjusted using the Benjamini and Hochberg’s approach to control the false discovery rate. The corrected *p-*value of 0.05 was used as the threshold to determine statistical significance. When the corrected *p-*value was < 0.05, the GO term was considered significantly enriched in the DEGs [20].

### Pathway analysis of DEGs

We used blastall (http://nebc.nox.ac.uk/bioinformatics/docs/blastall.html) to annotate the pathways of DEGs based on the Kyoto Encyclopedia of Genes and Genomes (KEGG) database. The enriched KEGG pathways were identified using the same formula as that used for GO analysis. *M* is the number of certain genes annotated to the specific pathways; *N* is the number of genes that were added with KEGG annotation; *m* represents the number of DEGs included in *M*; and *n* is the number of DEGs included in *N* [20].

### Quantitative real-time reverse-transcription PCR verification

Quantitative real-time reverse-transcription-PCR (qRT-PCR) was used to verify the expression levels of the DEGs identified by RNA-Seq analysis. The genes selected for validation by qRT-PCR were involved in enriched GO terms and enriched KEGG pathways. We designed the primers using Primer 5 software. The gene encoding β-actin of the Chinese mitten crab was selected as internal control to normalize the expression levels of the DEGs; all the experiments were conducted in triplicate. The PCR reaction was carried out in a 25 μL volume, comprising 0.5 μL of primers, 9.5 μL of RNase-free H_2_O, 12.5 μL of SYBR Premix Ex Taq, and 2 μL of cDNA. The thermal cycling program was as follows: 95°C for 30 s, followed by 40 cycles of 95°C for 5 s, 60°C for 30 s, and 72°C for 30 s. Melting curve analysis was performed at the end of qRT-PCR to verify the PCR specificity. The expression levels of DEGs were analyzed using the 2^—ΔΔ^ CT method [20].

## Results

### Illumina sequencing and quality assessment

RNA-Seq was performed using Illumina sequencing to investigate the transcriptional changes in *E*. *sinensis* with HPND. In the test groups, 48,219,524; 44,950,578; and 46,421,300 raw reads were obtained; and in the control groups 31,060,184; 31,016,354; and 32,478,004 raw reads were obtained. After the raw sequence reads were filtered and checked, we obtained 46,485,186; 43,436,726; and 44,832,770 trimmed reads in the test groups, and 30,506,938; 30,453,508; and 31,904,838 in the control groups. The trim rates of test groups were 96.40, 96.63, and 96.58%, and in the control groups were 98.22, 98.19, and 98.24%. The average length of the reads was 150 bp (Table 1). These results revealed that the transcriptome sequencing of the Chinese mitten crab was successful, and the trimmed reads of test and control groups could be used for the further analyses.

**Table 1:** Summary of reads obtained from *E*. *sinensis* transcriptome sequencing.

Group	Raw reads	Trimmed reads	Clean Q30 Bases Rate (%)	Average length (bp)	Trim rate (%)
Control 1	31,060,184	30,506,938	90.41	150	98.22
Control 2	31,016,354	30,453,508	90.90	150	98.19
Control 3	32,478,004	31,904,838	91.07	150	98.24
Test 1	48,219,524	46,485,186	91.53	150	96.40
Test 2	44,950,578	43,436,726	91.81	150	96.63
Test 3	46,421,300	44,832,770	92.14	150	96.58

### Comparison with the reference genome

We compared the trimmed reads with the reference genome sequence of the Chinese mitten crab [26]. The total mapped rates of the reads were 55.92, 56.24, and 56.20% in the three test groups and 58.82, 60.14, and 56.83% in three control groups. The numbers of multiple mapped reads for the three test groups and three control groups were 2,664,557; 2,522,319; and 2,708,791; and 1,353,719; 1,374,223; and 1,162,749, respectively, which accounted for 5.73, 5.81, and 6.04%; and 4.44, 4.51, and 3.64% of the total reads, respectively. The numbers of unmapped reads in the three test groups were 20,491,399; 19,008,186; and 19,634,582; and in the three control groups were 12, 562,134; 12,138,743; and 13,772,587 (Table 2).

**Table 2:** Statistical results for mapping the trimmed reads with the reference genome.

Map to genome		Total reads	Mapped reads	Unmapped reads	Multiple mapped reads
Control 1	Read numbers	30,506,938	17,944,804	12,562,134	1,353,719
	Percentage	100%	58.82%	41.18%	4.44%
Control 2	Read numbers	30,453,508	18,314,765	12,138,743	1,374,223
	Percentage	100%	60.14%	39.86%	4.51%
Control 3	Read numbers	31,904,838	18,132,251	13,772,587	1,162,749
	Percentage	100%	56.83%	43.17%	3.64%
Test 1	Read numbers	46,485,186	25,993,787	20,491,399	2,664,557
	Percentage	100%	55.92%	44.08%	5.73%
Test 2	Read numbers	43,436,726	24,428,540	19,008,186	2,522,319
	Percentage	100%	56.24%	43.76%	5.81%
Test 3	Read numbers	44,832,770	25,198,188	19,634,582	2,708,791
	Percentage	100%	56.20%	43.80%	6.04%

### Analysis of DEGs

**Fig 1:**
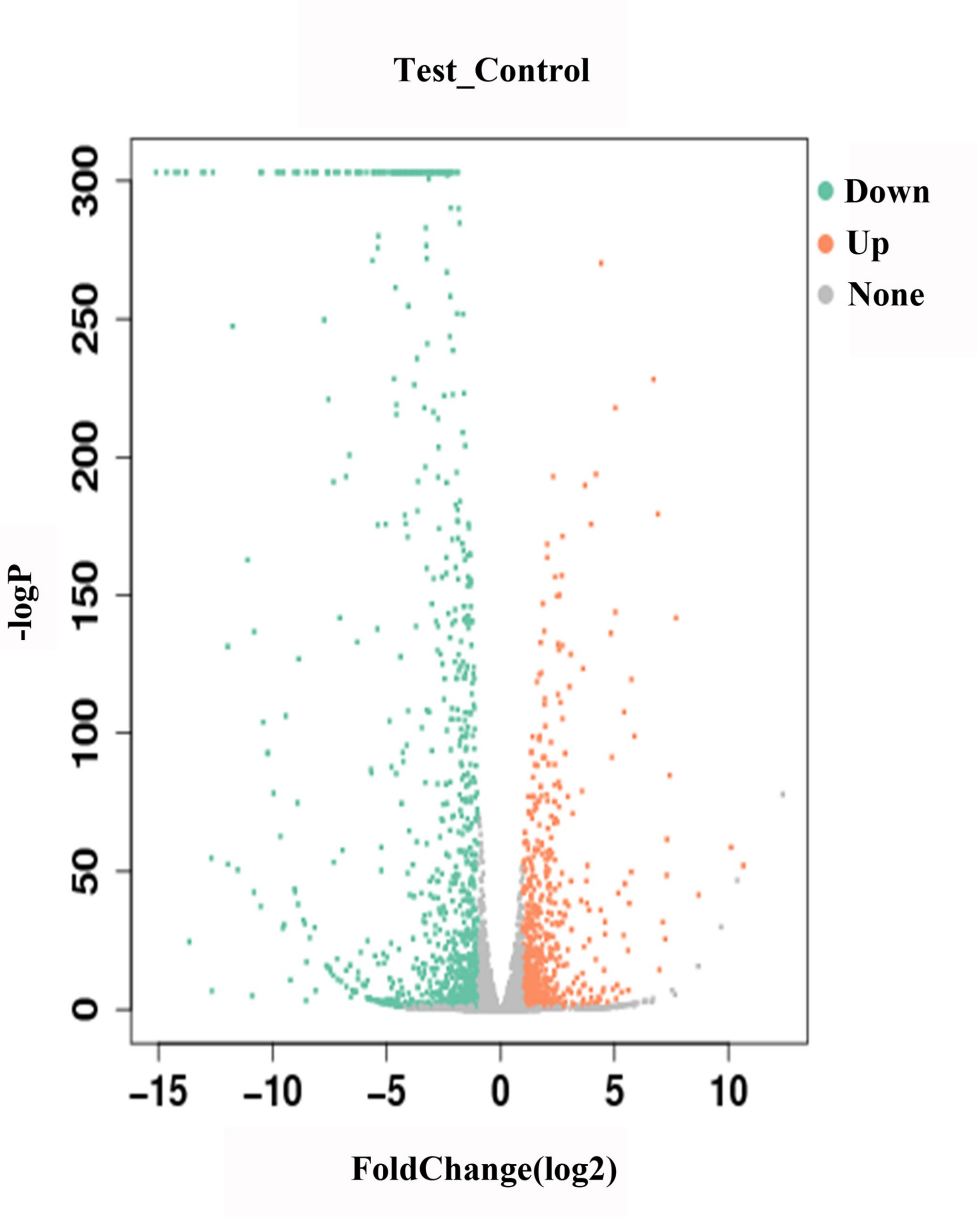
Volcano plot of the degree of differences in the expression profile of *E*. *sinensis* samples between the test and control groups. The x-axis is log2 (fold-change), and the y-axis is -log2 (p-value). Red represents significantly upregulated genes, green represents significantly downregulated genes, and every dot represents one gene.

To explore the DEGs of the Chinese mitten crab with HPND, we drew a volcano plot by comparing the differentially expressed genes (DEGs) of each group. The x-axis was the change in gene expression multiple in the different groups, and the y-axis was the statistically significant changes in gene expression (Fig 1). We identified 978 significantly downregulated DEGs and 644 significantly upregulated DEGs in the HPND group compared with those in the healthy group. The results showed that HPND changed the gene expression of the Chinese mitten crab.

### GO annotation of DEGs

**Fig 2:**
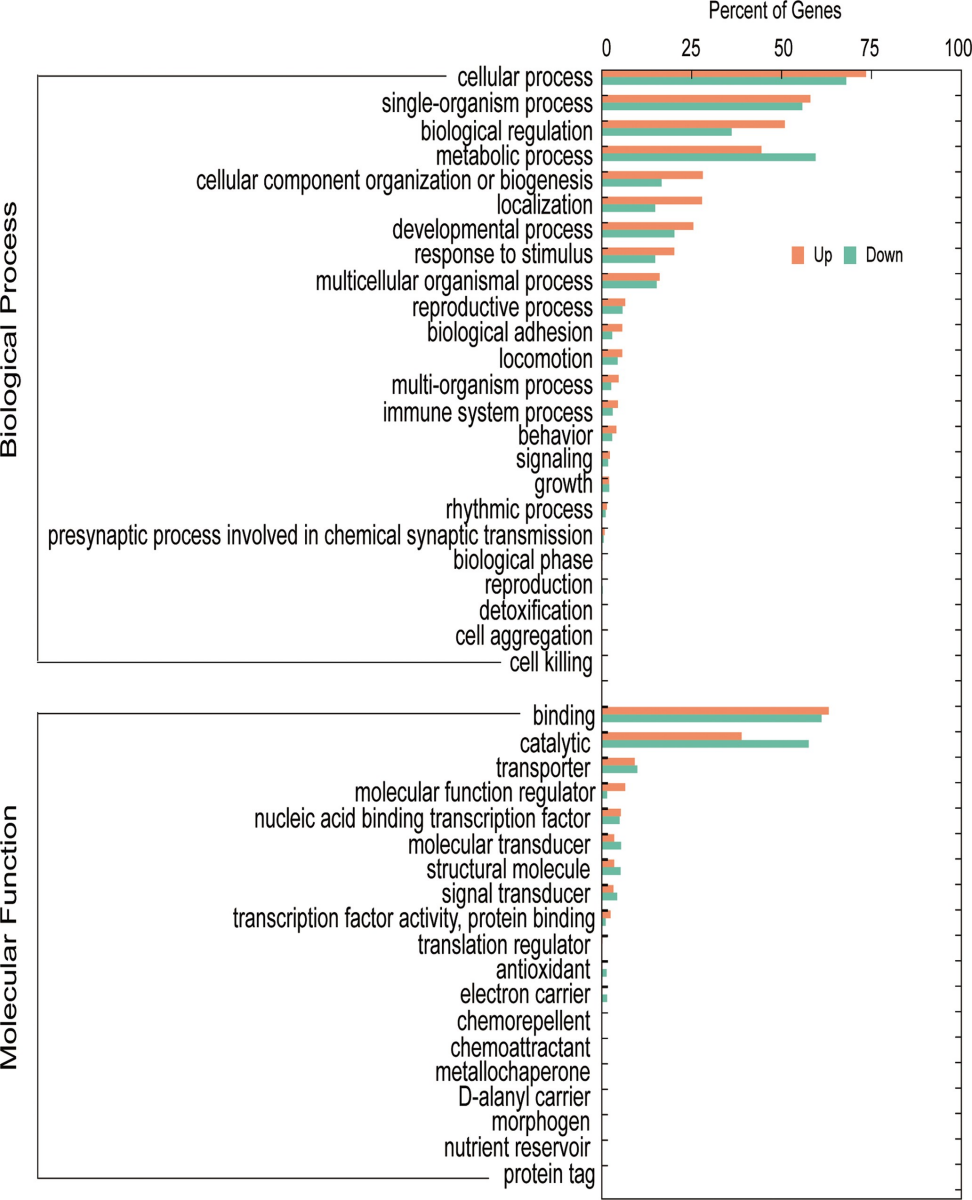
Histogram of enriched subcategories after gene ontology (GO) analysis of the DEGs in *E*. *sinensis* samples with HPND. The x-axis represents the percent of genes. The y-axis represents GO terms involved in the two main ontologies (biological process and molecular function).

The 1622 DEGs were classified into 65 GO terms belonging to the three domains: biological process, cellular component, and molecular function (Fig 2 and S1 Fig).

Lipids are stored in droplets in crabs and represent the major component of the fat body and the main source of metabolic fuel [27]. When et al. [28] reported that the energy of mature crabs is mainly derived from fat, followed by carbohydrates, with proteins providing the smallest proportion of energy. Research showed that prawns mainly used carbohydrates as a source of energy in response to stress [29]. When the existing carbohydrates are insufficient to provide the energy needed by the body, prawns changed their metabolic pathways to gain energy by breaking down fat and proteins [30, 31]. Under long-term stress, the catabolism of the body is strengthened and anabolism is weakened, resulting in the exhaustion of certain nutrients, which can not only disturb the normal physiological state of the body, but also damage the tissues and organs of the body [32, 33]. In the present study, we found that the expression levels of DEGs involved in “lipid metabolic process” and “carbohydrate catabolic process” were mostly downregulated in the GO annotation. These changes to the two energy metabolism processes in crabs might indicate that HPND in *Eriocheir sinensis* was related to stress of some kind.

### KEGG pathway analysis of DEGs

**Fig 3:**
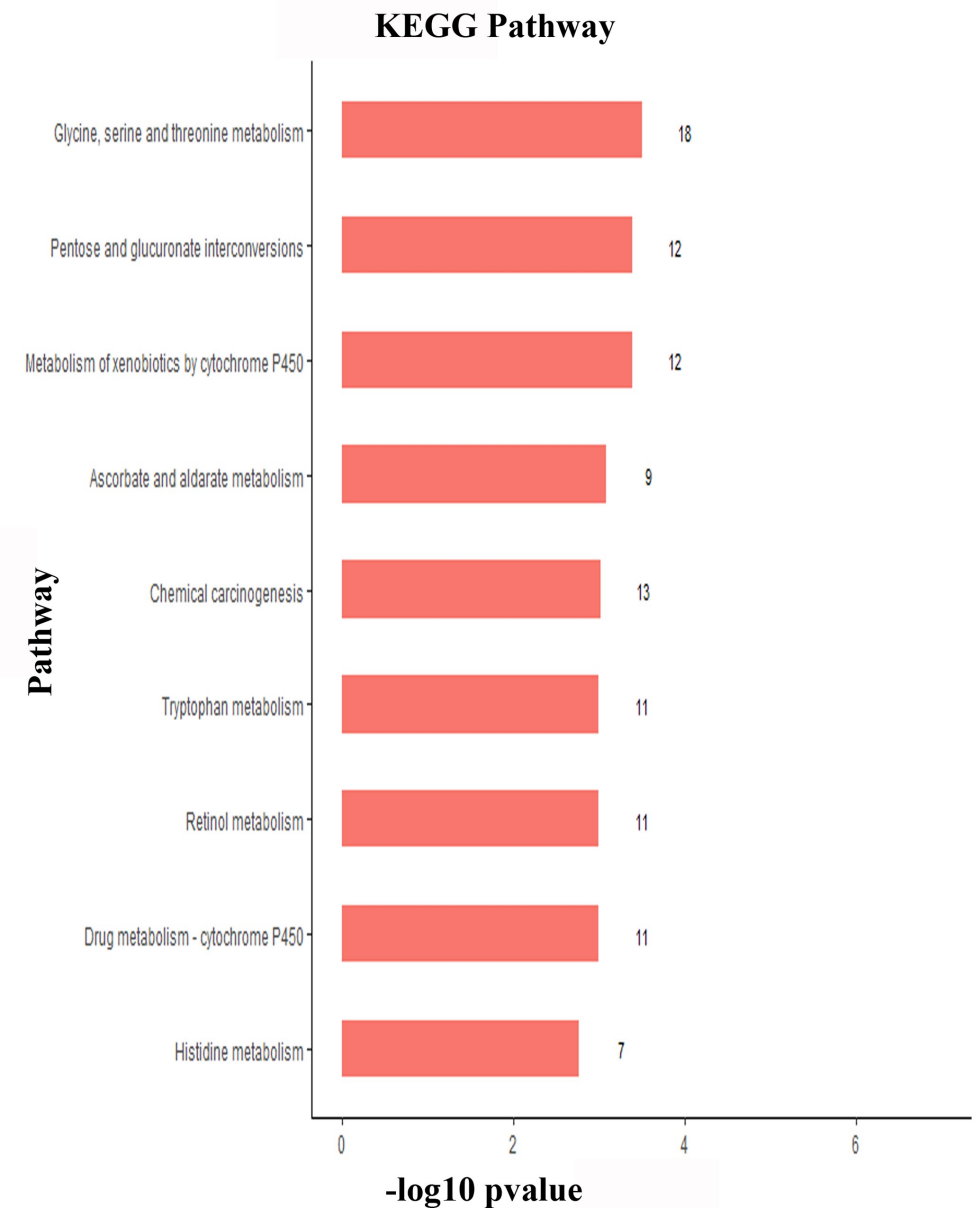
Histogram of the most enriched Kyoto Encyclopedia of Genes and Genomes (KEGG) pathways of DEGs in *E*. *sinensis* samples with HPND. The x-axis represents the statistical significance of the enrichment. The y-axis represents the KEGG pathway categories.

We mapped the DEGs to the KEGG database to further explore their related biological functions and important pathways. We found that some genes participated in multiple pathways, whereas other genes were included in a single pathway. The first nine most-enriched KEGG pathways of the DEGs were metabolism of xenobiotics by cytochrome P450; glycine, serine and threonine metabolism; pentose and glucuronate interconversions; drug metabolism—cytochrome P450; retinol metabolism; tryptophan metabolism; ascorbate and aldarate metabolism; chemical carcinogenesis; and histidine metabolism (Fig 3). Furthermore, we found that the downregulated DEGs were involved in the following pathways: Metabolism of xenobiotics by cytochrome P450; drug metabolism—cytochrome P450; and chemical carcinogenesis.

### Verification of the differential expression of DEGs

**Fig 4:**
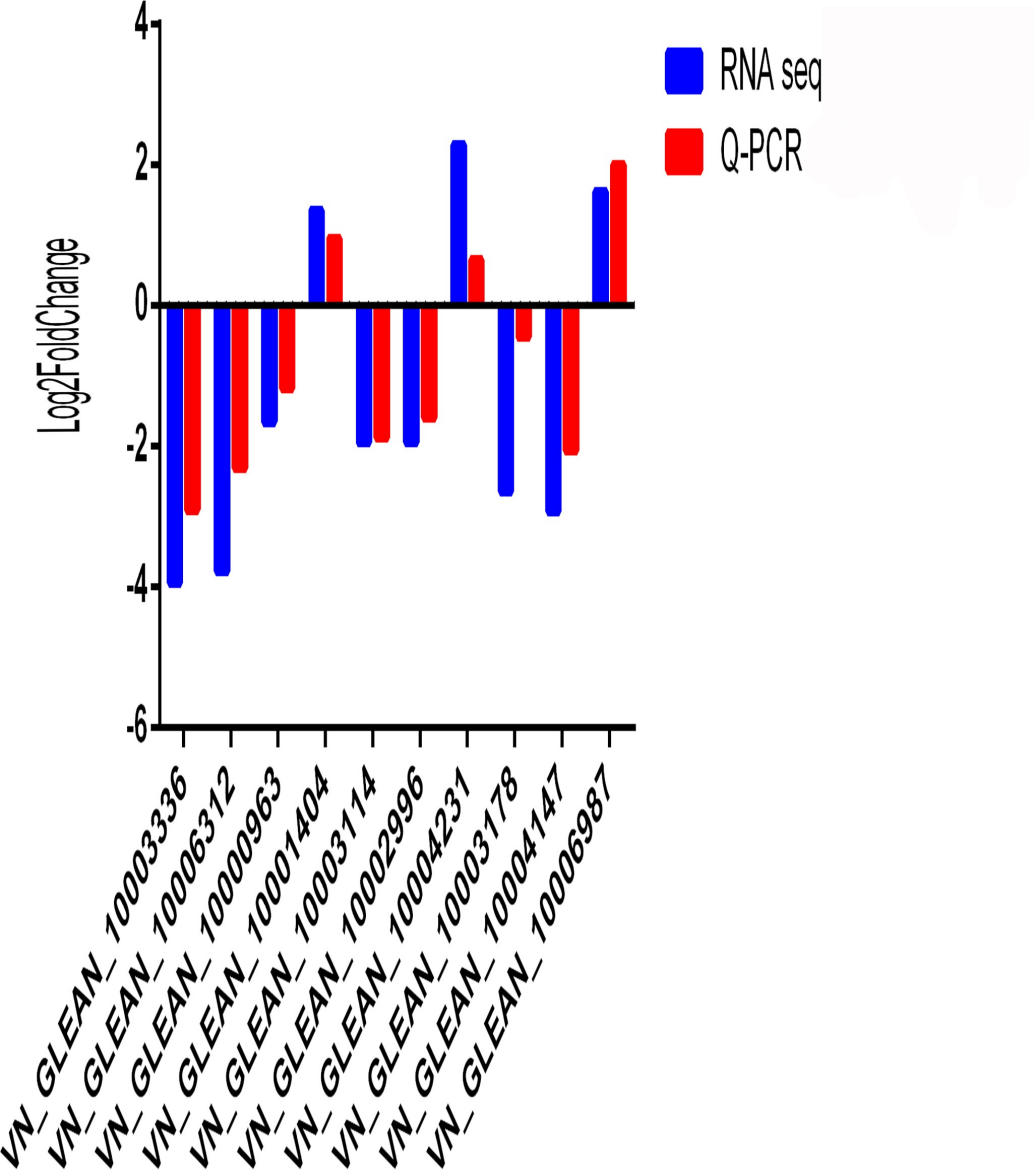
Comparison of 10 gene expression levels between the RNA-sequencing (RNA-Seq) data and the quantitative real-time reverse transcription PCR (qRT-PCR) data.

We used qRT-PCR to verify the gene expression profiles of 10 DEGs selected randomly after the GO and KEGG analyses. All the primer sequences of the 10 examined genes are listed in Table 3. The qRT-PCR results revealed no differences compared with the results of RNA-Seq for the 10 genes (Fig 4). These results further confirmed that the results of RNA-Seq were reliable.

**Table 3:** Oligonucleotide primers for qRT-PCR to validate DEGs expression.

Gene name	Predict function	Go category	Pathway name	Nucleotide sequence (5’-3’)	Expected product (bp)
ACTIN	—	—	—	F-TCACACACTGTCCCCATCTAG	114
				R- ACCACGCTCGGTCAGGATTTTC	
VN_GLEAN_10003336	Carbonyl reductase	carbonyl reductase (NADPH) activity (Molecular function)	Metabolism of xenobiotics by cytochrome P450	F-CACGGGCGATACTGAGAACC	109
				R- ATACGGAGGCGAATGAGACC	
VN_GLEAN_10003178	Fructose-bisphosphate aldolase class-I.	glycolytic process (Biological process)	Fructose and mannose metabolism	F-GGACCTCGTGAAGTTGAAGCA	119
				R- GGCGAAGACGCAGATGTTG	
VN_GLEAN_10006312	xanthine dehydrogenase-like isoform X1	oxidoreductase activity (Molecular function)	Drug metabolism—other enzymes	F-GGTGGTCTGGCTCTGATGTG	189
				R- CTCCGATCAGCGACAATCC	
VN_GLEAN_10000963	hypothetical protein	oxidoreductase activity (Molecular function)	Fatty acid degradation	F-GATGTGATGCTGACCAAGTGC	106
				R- CCAAATCCTGAGGCGATAGTG	
VN_GLEAN_10001404	AGAP000519-PA	lipid metabolic process (Biological_process	Glycerolipid metabolism	F-ATCTTATGTCTCAGCCCTCACG	178
				R- TGTTGCATATTCCTTGGTGTAGTG	
VN_GLEAN_10004147	ABC protein	organic anion transmembrane transporter (Molecular function)	ABC transporters	F-GGCTGGCATTGTAGGGACC	157
				R- GCCCACTCGGAACATTATCTG	
VN_GLEAN_10003114	putative aminopeptidase	catalytic activity (Molecular function)	Glutathione metabolism	F-GCCTGAGCATAGAGGGAAACC	200
				R- TTGTCAGCAACAGTCAGAGCC	
VN_GLEAN_10006987	AAEL009055-PA	cell surface (cellular component)	Cell adhesion molecules (CAMs)	F-GGTCACTCTTCTCAGGAAAATCTC	204
				R- GCATTGAACATTACTGTCCCATC	
VN_GLEAN_10002996	putative aminopeptidase	catalytic activity (Molecular function)	Glycolysis / Gluconeogenesis	F-AGTCAGCGTTGGCTCTATGTG	120
				R- GTGTTTGAGGAGGTCAGGGTC	
VN_GLEAN_10004231	uncharacterized protein	single-organism process (Biological process)	p53 signaling pathway	F-CGACGCAAGAAGAGTGACGA	169
				R- TAAGCAGGCTGAGGGGACC	

Positive values indicate that the expression of the DEG is upregulated. Negative values indicate that the expression of the DEG is downregulated.

## Discussion

The GO annotation showed that compared with those in healthy crab, the expression levels of most DEGs involved in the lipid metabolic process and carbohydrate catabolic process in crabs with HPND were downregulated. Furthermore, the KEGG analysis showed that DEGs participating in the biosynthesis of unsaturated fatty acids were downregulated, and the DEGs involved in glycerolipid metabolism were also downregulated, except for two genes (VN_GLEAN_10001404 and VN_GLEAN_10006205). In the enrichment analysis of KEGG pathways, “glycerolipid metabolism” and “biosynthesis of unsaturated fatty acids” were the important pathways related to lipid metabolism in *E*. *sinensis*, and the expression levels of genes involved in these two pathways were mostly downregulated. The results indicated that abnormal lipid storage and mobilization occurred in crabs with HPND. In addition to lipid metabolism, DEGs that were enriched in pathways including glycine, serine and threonine metabolism and pentose and glucoronate interconversions were mainly downregulated. These results were consistent with the results of previous studies, in which nutrients metabolic abnormalities, especially fatty acid and carbohydrate metabolism, were proposed to be associated with HPND in the Chinese mitten crab [8, 9].

In the present study, the enriched drug metabolic pathways associated with HPND in the Chinese mitten crab were metabolism of xenobiotics by cytochrome P450, drug metabolism-other enzymes, drug metabolism-cytochrome P450, chemical carcinogenesis, and glutathione metabolism. Interestingly, all the DEGs associated with these pathways were downregulated. Furthermore, we found that the expression levels of carboxylesterase family genes were upregulated, and CYP450 family genes were downregulated. Previous studies have showed that the biodegradation of pyrethroids was mainly catalyzed by carboxylesterases, P450 enzymes, and glutathione-*S*-transferase (GST) [34]. Carboxylesterase activity could be used as a reliable indicator of pyrethroids in the process of toxicity identification, and plays an important part in removing the toxicity of pyrethroids [35], CYP450 enzymes were inhibited by pyrethroids *in vivo* and *in vitr*o [36], and pyrethroids affect material metabolism [20]. Considering that previous research failed to find a relationship between HPND in the Chinese mitten crab and viral, bacterial, or other pathogenic organisms, we speculated that pyrethroids, which are widely used for pond cleaning [37] in Xinghua city, might cause HPND in the Chinese mitten crab.

## Conclusions

Our study conducted a comparative transcriptome analysis between diseased crabs with HPND and healthy crabs. GO functional annotation analysis and KEGG pathway analysis showed that abnormal metabolism of nutrients occurred in the diseased crabs. Alterations in the expression levels of CYP450 and carboxylesterase family genes, which are involved in xenobiotics or drug associated pathways, suggested that pyrethroids or other drugs were related to HPND in crabs.

In conclusion, our study revealed that under the stress effects of some as-yet-unidentified toxic substances, abnormalities in metabolism and absorption of nutrients appeared in crabs, which subsequently led to HPND. This hypothesis is consistent with that of Shen et al. [8]. However, whether the toxic substances were pyrethroids alone requires further study. Our research provide new information about the etiology and pathogenesis of HPND in the Chinese mitten crab, and further supported the hypothesis that environment factors play important roles in the formation of HPND in the Chinese mitten crab [38].

## Supporting information

S1 Fig Histogram of enriched subcategories after gene ontology (GO) analysis of the DEGs in *E*. *sinensis* samples with HPND. The x-axis represents the GO terms involved in the three main ontologies (biological process, cellular component, and molecular function). The y-axis represents the percent of genes.
(TIF)

S1 Table Differentially expressed genes. (XLS)

S2 Table GO analysis of the differentially expressed genes. (XLS)

S3 Table KEGG pathways of all unigenes. (XLS)

S4 Table Top nine most-enriched KEGG pathways of the differentially expressed genes. (XLS)

S5 Table Validation results of 10 differentially expressed genes using RT-PCR. (XLS)
