# Supplementary figures and images for: Transcriptome analysis of hepatopancreas of Eriocheir sinensis with hepatopancreatic necrosis disease (HPND)
Source: PLoS One. 2020 Feb 21;15(2):e0228623. doi: 10.1371/journal.pone.0228623 (PMC7034867; doi:10.1371/journal.pone.0228623)

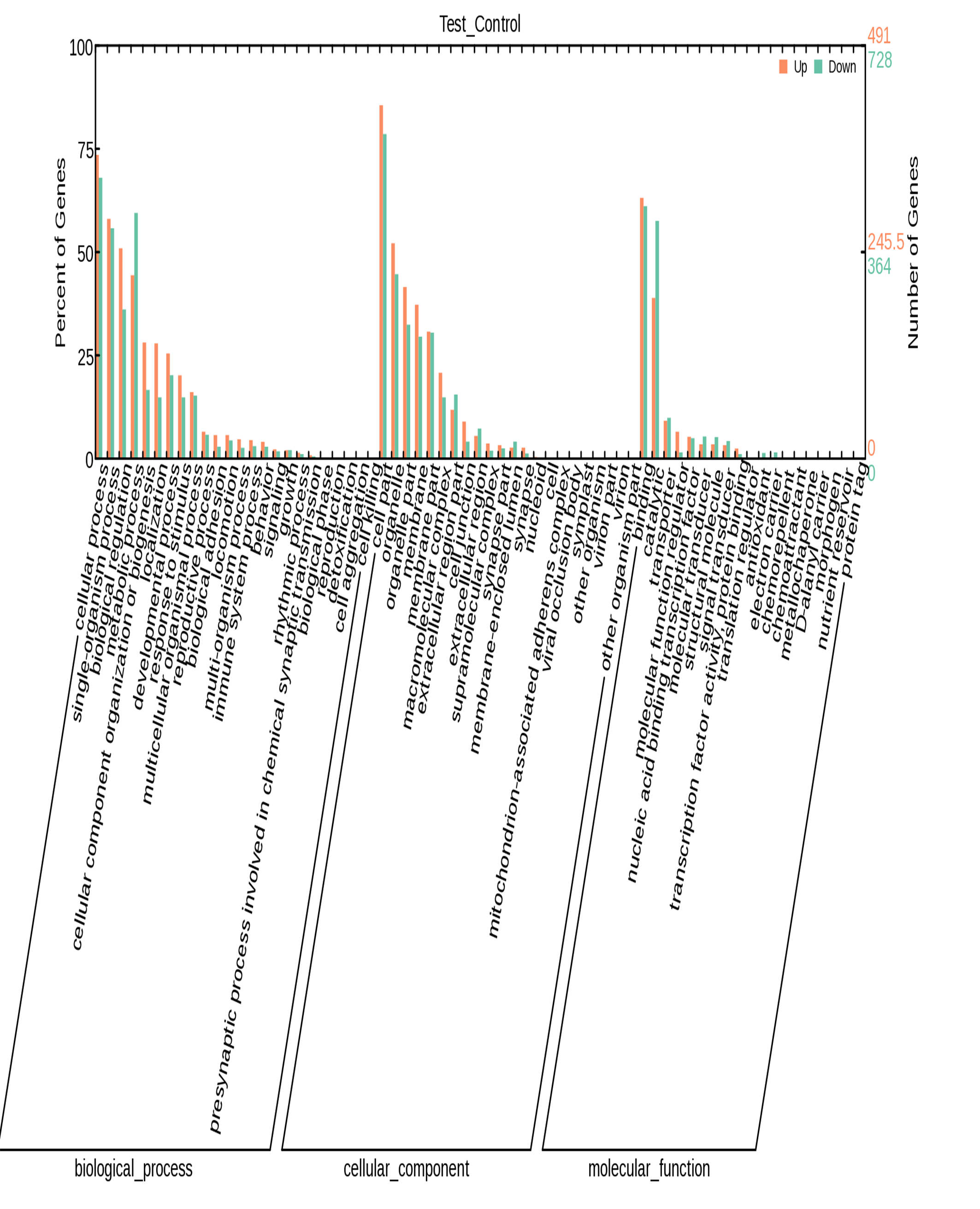

Supplement: S1 Fig — The x-axis represents the GO terms involved in the three main ontologies (biological process, cellular component, and molecular function). The y-axis represents the percent of genes. (TIF) [file pone.0228623.s001.tif]
